# Alkaline Phosphatase in Stem Cells

**DOI:** 10.1155/2015/628368

**Published:** 2015-02-12

**Authors:** Kateřina Štefková, Jiřina Procházková, Jiří Pacherník

**Affiliations:** ^1^Institute of Experimental Biology, Faculty of Science, Masaryk University, Kotlářská 2, 611 37 Brno, Czech Republic; ^2^Institute of Biophysics, Academy of Sciences of the Czech Republic, v.v.i., Královopolská 135, 612 65 Brno, Czech Republic; ^3^Department of Histology and Embryology, Faculty of Medicine, Masaryk University, Kamenice 5, 625 00 Brno, Czech Republic; ^4^International Clinical Research Center, Center of Biomolecular and Cellular Engineering, St. Anne's University Hospital Brno, Pekařská 53, 656 91 Brno, Czech Republic

## Abstract

Alkaline phosphatase is an enzyme commonly expressed in almost all living organisms. In humans and other mammals, determinations of the expression and activity of alkaline phosphatase have frequently been used for cell determination in developmental studies and/or within clinical trials. Alkaline phosphatase also seems to be one of the key markers in the identification of pluripotent embryonic stem as well as related cells. However, alkaline phosphatases exist in some isoenzymes and isoforms, which have tissue specific expressions and functions. Here, the role of alkaline phosphatase as a stem cell marker is discussed in detail. First, we briefly summarize contemporary knowledge of mammalian alkaline phosphatases in general. Second, we focus on the known facts of its role in and potential significance for the identification of stem cells.

## 1. Alkaline Phosphatase

Alkaline phosphatase (AP, EC 3.1.3.1 orthophosphoric-monoesterase, alkaline optimum) is a membrane bound enzyme which occurs in almost all living organisms. Mammalian APs have low sequence identity with* E. coli*, but residues involved in the active site of the enzyme and the ligands coordinating the two zinc atoms and the magnesium ion are conserved; thus, the catalytic mechanism is considered to be similar in prokaryotic and eukaryotic APs [1, 2]. Individual mammalian alkaline phosphatases differ in their heat stabilities and uncompetitive inhibition properties. This is the result of their nonhomologous disulphide bonds, their structural significance, and nonconserved residues [3, 4]. In humans, and probably also in other mammals, four isoenzymes have developed during evolution and are coded by up to four genes. These four isoenzymes can be found in various tissues, where they have different physiological functions. We know three isoenzymes of AP which are specific for certain tissues in humans; these are tissue-specific alkaline phosphatases (TSAP). They are intestinal alkaline phosphatase (IAP; Kasahara isoenzyme), placental alkaline phosphatase (PLAP; Regan isoenzyme), and germ cell alkaline phosphatase (GCAP; Nagao isoenzyme), which are expressed by embryonic cells but also carcinoma cells. The fourth isoenzyme is ubiquitous, and we call it tissue nonspecific alkaline phosphatase (TNAP). TNAP occurs in three isoforms which are bone, liver, and kidney TNAP [5].

In mice, there are also four APs; three of them are tissue specific and the fourth is tissue nonspecific. Intestinal, intestinal-like (IAP-like), and embryonic AP (EAP) are TSAP. The IAP-like AP was identified in mice, which have a knockout gene for IAP and still measurable residual activity in the gut. Murine EAP is similar to human GCAP [6]. Many important facts about the structure, physical and chemical properties, gene localisation, regulation, function, and tissue expression of APs can be obtained from Millán (2006) and McComb et al. (2011) [7, 8]. In the field of stem cell biology, TNAP are the main focus of interest, but EAP or GCAP, which are expressed in pluripotent inner call mass and primordial cells, respectively, cannot be ignored (see below). In general, TNAP and EAP/GCAP are frequently associated with germ layers, progenitors, and naive nondifferentiating cells.

In contrast, TSAPs are expressed with the increasing differentiation and maturation of particular cell lineages and tissues, because of their connection to the functions of such cells, for example, enterocytes (IAP) [9–11].

## 2. Expression of AP during Development

Similar to other lineages and developmental-specific genes recognized as determinants of stem cells, the expression pattern of AP during ontogenesis in some parts of tissues also corresponds with particular stem cell precursors and their niches. Thus, here, we briefly summarize the expression and presumable function of particular AP associated with cell differentiation potential (such as pluripotency, e.g.,) and stemness during development in general.

The expression of alkaline phosphatase can already be detected in 2-cell stage preimplantation embryos in mice and it is equally expressed in each embryonic cell to the stage of early blastocyst. At first, it is expressed by both the trophectoderm and inner cell mass (ICM). In the stage of late blastocyst, it seems that AP is strictly expressed in ICM. Lepire and Ziomek [12] described the isoenzyme of AP in the preimplantation stage of embryo-embryonic alkaline phosphatase (EAP). Until this discovery only two isoenzymes of AP had been defined in mice: EAP and TNAP. TNAP is also expressed in the preimplantation embryo, but 10 times less than EAP. Approximately 7 days* post coitum* (dpc) the expression of* Akp5* (EAP) decreases rapidly and* Akp2* (TNAP) becomes the major gene of AP, which is expressed between 7–14 dpc in primordial germ (PG) cells [6]. The PG cells exhibit high activity of TNAP during migration to the developing gonad. The activity of TNAP decreases 14-15 dpc in these cells [13]. In humans, it is known that the expression of alkaline phosphatases is detectable prior to 4 weeks of gestation [14, 15].

In contrast to mice PG cells (which express TNAP), GCAP activity is observed in human migrating PG cells [15–17]. In the adult, it is primarily synthesized in the testes, cervix, and thymus. Trace amounts are synthesized in placenta and lung tissues [18]. It is not known whether AP is expressed in the preimplantation stage of human embryos. Also, little is known about the expression of other isoenzymes in human embryonic development because of ethical limitations. On the 8th day* post coitum* (in mice), TNAP is also expressed in the neuroectoderm [19]. Later (9.5 dpc), TNAP activity is observed in the area of the brain and spinal cord. Between 10.5 and 14.5 dpc, TNAP activity is observed in the mesencephalon and the rhombencephalon, along the entire spinal cord and cranial nerves, and at the end of this stage TNAP positive fibres are in the pons. Fourteen-and-a-half days* post coitum*, TNAP expression is decreased in the neuroepithelium. In the adult, TNAP positive clusters are observed in the subependymal layer, where the neural progenitors are localized. It is suggested that TNAP is involved in the development of the nervous system partly as an ectonucleotidase in neurogenic zones [20, 21] or specifically by interacting with collagen during neuronal migration [22, 23]. However, TNAP activity is detectable in the choroid plexus up to adult age [23].

However, amongst all vertebrates (including humans), TNAP activity is dominant in the developing skeleton, because TNAP is involved in the mineralization of tissues. The activity within the developing skeleton is associated with the expression of TNAP in chondrocytes and osteoblasts. In mice this arises between 13 and 14 dpc [19].

The expression of another AP, TSAP, is also mostly associated with cell differentiation and such AP activity is generally recognized as a marker of cell differentiation [9, 24–26].

## 3. Role and Function of AP

If we ask why some stem cells express AP, we must know how APs are utilized by particular cells. In general, AP catalyzes the hydrolysis of phosphate esters. AP exhibits three types of activity [27–31]:

1. hydrolytic activity R–P + H–OH→R–OH + H–P,
2. phosphotransferase activity R–P + R′–OH→R–OH + R′P,
3. pyrophosphatase activity R–P–P–R′+ H–OH→R–P + R′–P.

Hydrolytic activity is considered a general reaction which is catalysed by AP.

The role of individual APs is also apparent from the phenotype of organisms with nonfunctional AP. EAP depletion is not associated with any detectable abnormalities. Also, the depletion of TNAP has no effect on the differentiation or migration of PGC, so we may suggest a similar state also for GCAP in human PGC; however, experimental data are unobtainable due to ethical limitations. PLAP, which is only known in primates, enables the transport of maternal IgG through the placenta and, by an unknown mechanism, improves the growth and development of embryos and cells in general. High levels of GCAP and PLAP are also markers of tumor diseases, typically, such neoplasia as germ cell tumours [32, 33], squamous cell carcinoma of the lung [34], and carcinoma of the gastrointestinal tract and uterus [35, 36].

The key roles of TNAP are in the mineralization of hard tissue (it provides free phosphate for the creation of hydroxyapatite crystals and hydrolyses pyrophosphate, an inhibitor of bone matrix formation) and in the metabolism of vitamin B6, and thus in the metabolism of neurotransmitter *γ*-aminobutyric acid (GABA). Therefore, the depletion or recessive mutation of TNAP leads to defects in both the mineralization of hard tissue and the development of the nervous system.

IAP plays a role in the transport of fatty acids and triglycerides from the intestinal tract to the circulation [37–40]. IAP also regulates duodenal surface pH [41] and detoxifies bacterial endotoxins in the colon by means of dephosphorylation [42, 43].

## 4. Regulation of the Expression and Activity of AP

AP activity clearly correlates with its expression, and its fine regulation is mediated by the actual microenvironment rather than by some individual signaling pathways. Thus, AP expression and activity are regulated mainly through the developmental status of cells or tissues. Therefore, as mentioned above, AP expression is a generally suitable marker of differentiation processes both* in vivo* and* in vitro* for particular cell types. Although some data on the role of p38 kinase (mitogen-activate protein kinase (MAPK) p38) in the regulation of TNAP expression exist, the precise mechanism remains unknown [44–47]. Similarly, we observed both a decreased level of AP and decreased AP activity in p38 −/− ES cells [48, 49] in comparison with their wt counterparts, while the expressions of pluripotent markers such as Oct-4, Nanog, and Zfp42 remained unchanged (our unpublished data).

## 5. Alkaline Phosphatase and Stem Cells

### 5.1. Pluripotent Stem Cells

A high level of AP and high AP activity are traditional markers of pluripotent embryonic stem (ES) cells, both mouse and human. This is based on the fact that ICM is highly positive for AP activity, in contrast to trophoblast cells at the blastocyst stage. As ICM is committed to lineage differentiation, AP expression is downregulated and it appears in discrete specialized cell populations such as PG cells and later also in other tissues, for example, in osteoblasts (see above). High AP activity is associated with the majority of pluripotent stem cells. Embryonal cancer (EC also called teratocarcinoma stem cells), embryonic germ (EG), the already mentioned embryonic stem (ES), and induced pluripotent stem (iPS) cells express high activity of AP. Interestingly, the absence of AP activity has been reported in pluripotent epiblast stem (EpiS) cells, which are derived from epiblasts of later developmental stages of the embryo than those from which ES cells are derived. The pluripotency of EpiS cells is partially restricted in comparison with other pluripotent stem cells, which correspond to their more differentiated phenotype, compared to ES cells [50, 51].

Mouse ES cells are derived from pluripotent cells of ICM of early blastocyst 3.5–4 dpc. At this time, the* Akp5* gene (coding EAP) is dominantly expressed in the embryo. However, mouse ES cells express the* Akp2* gene (coding TNAP), which is used for determination of their undifferentiated state [52]. Mouse PG, EG, and EC cells derived from teratocarcinoma also show high activity of TNAP [13, 53, 54]. The dominant expression of TNAP but not EAP in mouse ES cells supports the hypothesis concerning a shared predecessor of ES and PG/EG cells [55]. On the other hand, the shift in expression of AP isoenzymes may also correspond to the fact that the expression of AP is important for cells, but that, as cell environments are modified during developmental processes, particular AP may work more effectively in new conditions. This, we may suggest, is based on the sensitivity of particular AP to various amino acids and small peptides, which were determined as inhibitors of AP activity; these are presented in Table 1 [5, 56–62]. However, further experiments will be required to verify such a hypothesis.

In human embryos, GCAP activity is found in PG cells, in contrast to human ES cells, where TNAP is detected [63, 64]. However, it is uncertain whether GCAP is also expressed, which might be expected. Human EC cells express both isoenzymes, TNAP and GCAP [65]. In human EG cells, the GCAP isoenzyme is expected, because GCAP is expressed in migrating human primordial germ cells and gonocytes [18, 63]. However, precise studies of this phenomenon in humans are lacking.

In addition, some experimental results point to greater similarity between mouse ES/PG/EG/EC and human PG/EG/EC cells, but not human ES cells, which raises another question about the significance and role of AP in these cells [66].

However, this inconsistency in the phenotype of these cells was partially resolved by Brons et al. [51] and Tesar et al. [50]. Both these groups described so-called epiblast stem (EpiS) cells, which correspond to pluripotent cells of epiblast. Mouse EpiS cells are more differentiated than mouse ES cells. Mouse and human ES cells should be equivalent in their properties. However, human ES cells correspond more closely to mouse EpiS cells. Interestingly, EpiS cells do not exhibit detectable AP activity [50]. Thus, we can suggest that the precise determination of AP isoform expression patterns in human ES in comparison to the abovementioned cell populations may further improve our picture of its identity, regulation of self-renewal, and phenotype.

The specific importance of AP for pluripotent cells as well for pluripotent stem cells remains questionable. Andäng et al. suggest the importance of GABA synthesis in ES cells for the regulation of their proliferation and self-renewal [67] and that AP participates in GABA synthesis (see above). We may also suggest the increased need for substrate dephosphorylation associated with the quickly proliferating metabolism of ES cells. In ICM and other types of pluripotent cells, we can also hypothesise a similar role for AP as in ES cells.

Interestingly, as mentioned above and expected in EpiS cells, all known mouse and human pluripotent stem cells express TNAP preferentially. The expression of TNAP is also quickly upregulated during the process of reprogramming somatic cells into iPS cells. In the original protocol, the iPS phenotype is induced through the exogenous expression of four genes: Oct-4 (Pouf5), Sox-2, and Klf4, which are responsible for the maintenance of pluripotency, and c-myc, responsible for the induction and/or increasing rate of proliferation [68]. TNAP expression increases directly after the transfection of somatic cells by these four genes. This corresponds with the conception of the direct regulation of TNAP expression by Oct-4 and Sox-2 [69]; see Table 2. Thus, TNAP expression appears long before real cell reprograming and the reexpression of endogenous genes for Oct-4 or Sox-2 [70]. Further, our* in silico* analysis identified several binding sites for both Oct-4 and Sox-2 and further factors associated with pluripotency such as Nanog, Tcf3, Sa4b, and FoxD3 in promoters of TNAP (Table 2). For all the above-mentioned facts, AP activity is a widely accepted marker of pluripotent stem cells and it has been shown that the maintenance of AP activity in AP-positive colony formation positively correlates with the clonogenic and self-renewal potential of undifferentiated human ES cells in cultures [64]. In addition, AP activity is downregulated reciprocally with differentiation processes involving pluripotent stem cells [71]. On the other hand, depletion of the TNAP gene* Akp2* has no effect on the formation of either ICM or PG cells or their expansions in mouse [52]. Surprisingly, at the same time, both ICM and PG cells may be considered at least precursors of pluripotent stem cells, as are ES or EG cells. Unfortunately, little is known about the inability to isolate ES cells from TNAP−/− embryos. More importantly, a consequence of TNAP depletion is a negative effect on bone development and vitamin B turnover (see above).

Interestingly, although EAP is the dominant form of AP in pluripotent ICM, we recognized only one Nanog binding site within the mouse EAP gene, and no binding sites were observed for the other pluripotent genes mentioned above. In human GCAP, one binding site for the mentioned pluripotent genes, apart from FoxD3, was recognized (Table 2). The mechanism concerning the shift from EAP/GCAP expression in pluripotent ICM to TNAP expression in the other pluripotent cells and PG cells is still not understood.

However, it seems clear that TNAP expression is not closely associated with pluripotent stem cells or with pluripotency alone, because ICM cells express another AP dominantly and they have no pure self-renewal potential (and such ICM cells cannot be recognized as stem cells) [19]. Furthermore, PG cells are other cells which strongly express TNAP and other so-called pluripotent markers (Oct-4, Nanog), but they are not pluripotent.

Paradoxically, the regulation of TNAP expression in pluripotent stem cells is also influenced by the effect of retinoic acid (RA). RA has a prodifferentiation and pleiotropic effect on many cells both* in vivo* and* in vitro* [72, 73]. The RA effect is fast and strong. Cells undergo the differentiation process after the addition of RA to a culture independently of other conditions. The final phenotype depends on the RA concentration, type of culture (adherent, suspension), and presence of particular signaling molecules [72, 74–77]. It is interesting that TNAP activity increases with this differentiation, although both stemness and pluripotent markers decrease. This has been proven in both EC and ES cells ([78, 79] and Figure 2). The upregulation of TNAP expression is clearly mediated by RA receptors binding motifs in the TNAP gene (Table 2). The role and significance of this RA-induced TNAP expression in EC and ES cell differentiation are unknown. However, this observation goes against the hypothesis concerning the role of TNAP in fast proliferating cells. RA induces a decrease in the proliferation and accumulation of cells in the G1 phase of the cell cycle in both ES and EC cells [80–82].

Further, the* in vitro* study of the differentiation of ES cells to PG cells yields a remarkable observation. RA is required for the induction of PG cell formation during the EBs-mediated differentiation of ES cells [83]. In this system, 5-day-old EBs, in which TNAP, Oct-4, and Nanog expression have been downregulated, are treated by RA. After this treatment, PG cells can be determined within EBs. However, PG cells also express high levels of Oct-4, Nanog, and TNAP [84]. Increased TNAP may simply be explained by the presence of RA receptor (RAR/RXR) binding sites in the TNAP gene, as mentioned above. However, the expression of Oct-4 is directly inhibited by RA [85]. The expression of Nanog, the second most important pluripotent gene, can be positively regulated by RA, as has been shown in some recent studies [77, 86]. In this case, Nanog expression is probably induced through the induction of insulin-like growth factor (IGF) expression by RA or other retinoids [86]. IGF is a strong inductor of the PI3-K pathway, whose activity leads to the increasing expression of Nanog in ES cells [87, 88]. Thus, RA mediates the auto- and paracrine stimulation of PI3-K pathways by IGF. Importantly, Nanog itself regulates the expression of Oct-4 [89]. Thus, we suppose that, in the mentioned model of the RA-induced formation of PG cells, Oct-4 expression is upregulated and maintained by Nanog. The feedback in the regulation network Oct-4/Nanog and the positive role of IGF in the maintenance of ES cells are well known [87, 89, 90]. However, the factors that enable the above supposed RA-induced creation of Oct-4 positive cells (PG cells) in contrast to the fast differentiation of RA-treated pluripotent stem cells are still unknown. We can hypothesize a small degree of balance between the expression of RA-targeted genes and modulation of the Oct-4/Nanog network. The reciprocal ordering of Oct-4 and Nanog expression during the establishment of their network may also play an important role here. Recently, the preferential role of Nanog in PG cell formation rather than in pluripotency itself has also been demonstrated [91].

### 5.2. Other Stem Cells

**Figure 1:**
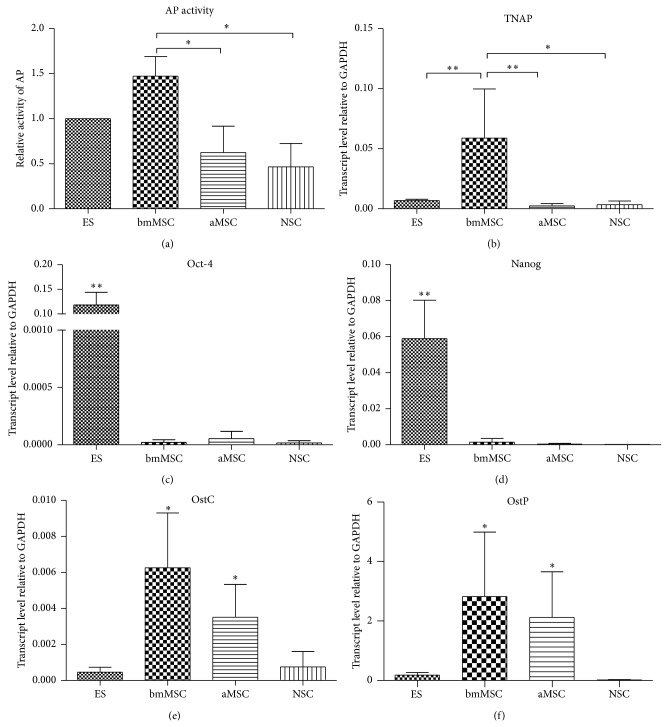
Relative alkaline phosphatase activity (a) and mRNA expression of TNAP ((b) qRT-PCR) in various stem/progenitor cells. Bone marrow-derived MSC (bmMSC) has both high AP activity and TNAP expression, followed by ES cells. Relative expression (qRT-PCR) of common pluripotent (Oct-4 (c) and Nanog (d)) and osteogenic (Osteocalcin, OstC (e) and Osteopontin, OstP (f)) genes. A high level of Oct-4 and Nanog documented the real pluripotent status of ES cells. A higher expression of OstC and OstP mRNA in bmMSC documented their osteogenic properties/lineages specification. (ES: mouse embryonic stem cells; NSC: neural stem/progenitor cells; bmMSC: bone marrow mesenchymal stem cells; aMSC: adipose tissue mesenchymal stem cells). Details of the presented assay may be found in our previous work [9, 77]. Primers and conditions for OstC and OstP were as follows: OstC: 5′-CTTGGGTTCTGACTGGGTGT-3′, 5′-GCCCTCTGCAGGTCATAGAG-3′ (212 bp, 60°C); OstP: 5′-TCACCATTCGGATGAGTCTG-3′, 5′-ACTTGTGGCTCTGATGTTCC-3′ (436 bp, 60°C). Error bars indicate ±SD (*n* ≥ 3, ^*^ *P* < 0.05; ^**^ *P* < 0.01, ANOVA post hoc Bonferroni's Multiple Comparison Test).

**Figure 2:**
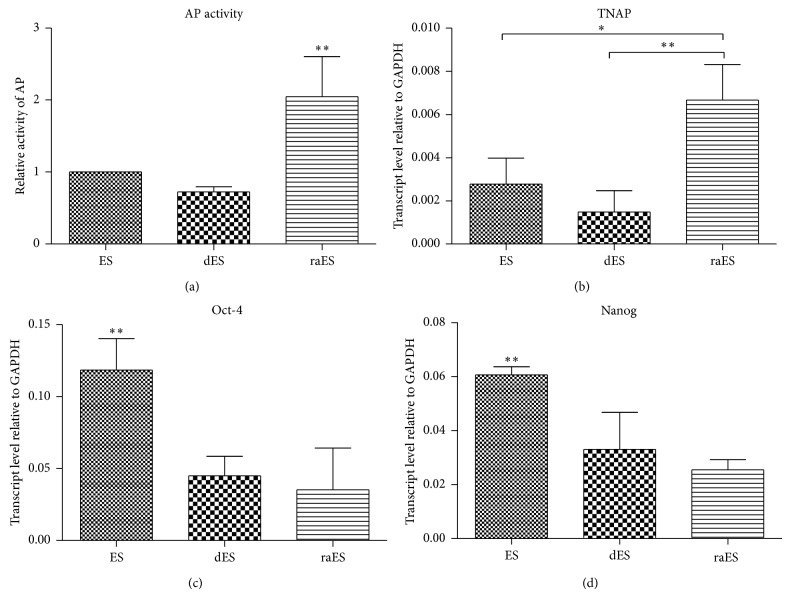
Retinoic acid increased AP activity (a) and mRNA expression of TNAP ((b) qRT-PCR) in mouse ES cells. On the other hand, retinoic acid downregulated the expression of pluripotent markers Oct-4 (c) and Nanog (d) in the same manner as the spontaneous differentiation of ES cells through leukemia inhibitory factor withdrawal [75]. (ES: mouse embryonic stem cells; dES: spontaneously differentiating ES cells for two days; raES: retinoic acid-treated (0.2 *μ*M) ES cells for two days). Details of the presented assay are as in Figure 1. Error bars indicate ±SD (*n* = 4, ^*^ *P* < 0.05; ^**^ *P* < 0.01, ANOVA post hoc Bonferroni's Multiple Comparison Test).

The high expression/activity of AP in ES cells leads to the presumption that it is a universal marker of SC. Recent research has not confirmed the accuracy of such an assumption. Except for spermatogonia stem cells, a high level of AP has not been definitively demonstrated as a general SC marker in other recognized tissue-specific stem (embryonal or adult) cells or stem cell rich populations. Nevertheless, Langer et al. [20] determined some TNAP positive cells in embryonal and adult central nervous systems. These neural TNAP positive cells were observed within cell populations of the subventricular zone, which is rich in neural stem cells and various neural progenitors. Some of these progenitors were TNAP positive. Here, TNAP was not associated with particular progenitors, but with subpopulations of several identified progenitors. It seems that TNAP expression in the subventricular zone (SVZ) is not associated with a specific cell phenotype, but with cell behaviour, for example, proliferation or migration [20]. This hypothesis is supported by the mitogenic effect of adenosine generated by TNAP through the hydrolyzation of nucleoside triphosphates and diphosphates [20, 92]. Further examples of AP activity in somatic stem cells can be found in reports of various sets of mesenchymal stem cells (MSC) [93–95]. Sobiesiak et al. [96] identified TNAP as a stemness marker of MSC, signed as MACS1. Recently, however, Kim et al. [97] presented proosteogenic properties of MSC with higher TNAP activity. MSC derived from adipose tissue exhibit significantly lower levels of TNAP transcript and activity (Figure 1). This is in contrast to the situation in pluripotent stem cells, in which it may be suggested that improved stemness correlates with high TNAP activity, excluding RA effects (see above). The notion that TNAP in mesenchymal cells is a mark of progenitors rather than MSC themselves is also supported by further studies documenting high TNAP in differentiated osteoblasts and odontoblasts [97, 98] (Figure 1). In addition, side population (SP) cells derived from human dental pulp tissue, which are rich in stem cells, exhibit lower levels of TNAP mRNA than the major population [98]. However, further detailed study in this field and on tissue-derived SP cells would be useful. Data comparing the expressions and activities of TNAP in various stem cell-enriched populations are presented in Figure 1.

## 6. Conclusion

TNAP expression is a suitable marker of pluripotent stem cells, but with some limitations. A high level of AP correlates very well with pluripotency and undifferentiated pluripotent stem cell phenotypes. A low level of AP activity denotes the restriction of pluripotency, which was also observed in EpiS cells [50, 51] and with differentiation. Some other limitations are mentioned above. In all other cases, that is, in adult SC, and so forth, a high level of AP is associated with the process of differentiation rather than with stemness. Thus, particular cell types are able to regulate the expression of TNAP, and probably also other APs, through various combinations of transcriptional regulatory networks. Accordingly, AP such as TNAP, for example, may be expressed under the control of Oct-4 in pluripotent cells and also in Oct-4 negative cells, such as mesenchymal cells and their progeny. Unfortunately, the importance of AP activity for pluripotent cells and/or stem cells (principally for pluripotent stem cells) is still generally unclear. Similarly, we do not understand the role of the shift in expression of EAP/GCAP to TNAP between ICM and ES cells. Does it play a role in the pluripotency of stem cells or does it only represent a marker of the common ancestor/precursor of ES and PG cells? Curiously enough, no PG or ICM cells are considered genuine pluripotent stem cells. PG cells are not pluripotent and both ICM and PG cells have limited self-renewal potential [55]. To answer the question of whether AP/TNAP level and activity are common markers of pluripotent stem cells, further work is required.

## Figures and Tables

**Table 1:** Alkaline phosphatases expressed in humans and mice with their basic attributes.

Isoenzyme AP	Gene	Molecular weight	Tissue localization		Temperature of inactivation	Specific inhibitors
PLAP	*ALPP *	90–120 kDa	**Syncytiotrophoblast**		Stable at 70°C	neuraminidase [5], L-phenylalanine [58], L-leucine [60], L-leucyl-glycyl-glycine [56], L-phenylalanyl-glycyl-glycine [56]
GCAP	*ALPPL2 *		**PGC**, **testes**, **cervix**, **thymus**		
EAP (mouse)	*Akp5* (*Alppl2*)		**Preimplantation stage of embryo**		

IAP IAP-like (mouse)	*ALPI* (human)* Akp3* (mouse) *Akp6* (*Alpi*)	70–90 kDa	**Small intestine** (duodenum)		Stable at 56°C	L-Phenylalanine [58], L-tryptophan [59]

Bone isoform TNAP	*ALPL *(human) *Alpl *(mouse)	120–150 kDa	**Bone** (preosteoblastic cells, basolateral site of osteoblasts), **chondrocytes**, **odontoblasts**	**PGC** (mouse), **brain **(subependymal layer), **embryo**,** testes** (mouse)	Nonstable at 55°C	L-Homoarginine [61], neuraminidase [5], L-levamisole [57], imidazole [62]
Liver isoform TNAP			**Liver **(bille epithelium)		At 55°C more stable than bone isoform	
Kidney isoform TNAP			**Kidney **(epithelial cells of proximal tubules)		Nonstable at 45°C	

PLAP: placental AP; GCAP: germ cell AP; EAP: embryonic AP; IAP: intestinal AP; TNAP: tissue nonspecific AP.

**Table 2:** Transcription factor binding site (TFBS) analysis of promoters of tissue nonspecific alkaline phosphatase isoforms.

Species	Gene symbol (name)	Matrix family/matrix					
		V$HOXF	V$STEM		V$SORY	V$FKHD	V$RXRF
		V$NANOG.01	V$OCT3_4.02 (Oct-4)	V$OSNT.01	V$SOX2.01	V$HFH2.01 (FoxD3)	V$RAR_RXR.01
Human	ALPP (PLAP)	1	—	—	—	—	2
	ALPPL2 (GCAP)	—	1	1	1	—	1
	ALPL (TNAP)	1	3	4	—	—	3

Mouse	Alppl2 (EAP)	1	—	—	—	—	—
	Alpl (Akp2/TNAP)	1	6^*^	2	1	1	7

NANOG, Oct3-4, SOX-2, FoxD3, and RAR/RXR binding motifs were predicted by the Genomatix software tool MatInspector (1) (digits denote numbers of matches) and further analysed using the rVista algorithm (based on Transfac Professional Library v10.2) (2) for evolutionary conserved motifs between mouse and human (grey fields). The asterisk “∗” symbol indicates previously observed Oct-4 binding in Alpl (Akp2) regulatory sequences using the Chip-PET methodology (3, 4). Matrix V$OSNT.01 represents composed binding sites for Oct-4, Sox-2, Nanog, Tcf3 (Tcf7l1), and Sall4b in pluripotent cells.

## References

[1] Kim E. E., Wyckoff H. W. (1991). Reaction mechanism of alkaline phosphatase based on crystal structures. Two-metal ion catalysis. *Journal of Molecular Biology*.

[2] Le Du M. H., Stigbrand T., Taussig M. J., Ménez A., Stura E. A. (2001). Crystal structure of alkaline phosphatase from human placenta at 1.8 Å resolution: implication for a substrate specificity. *The Journal of Biological Chemistry*.

[3] Kozlenkov A., Manes T., Hoylaerts M. F., Millán J. L. (2002). Function assignment to conserved residues in mammalian alkaline phosphatases. *The Journal of Biological Chemistry*.

[4] Le Du M.-H., Millán J. L. (2002). Structural evidence of functional divergence in human alkaline phosphatases. *The Journal of Biological Chemistry*.

[5] Moss D. W. (1982). Alkaline phosphatase isoenzymes. *Clinical Chemistry*.

[6] Hahnel A. C., Rappolee D. A., Millan J. L. (1990). Two alkaline phosphatase genes are expressed during early development in the mouse embryo. *Development*.

[7] Millán J. L. (2006). *Mammalian Alkaline Phosphatases*.

[8] McComb R. M., Bowers G. N., Posen S. (2011). *Alkaline Phosphatase*.

[9] Kovaříková M., Pacherník J., Hofmanová J., Zadák Z., Kozubík A. (2000). TNF-alpha modulates the differentiation induced by butyrate in the HT-29 human colon adenocarcinoma cell line. *European Journal of Cancer*.

[10] Krejčová D., Procházková J., Kubala L., Pacherník J. (2009). Modulation of cell proliferation and differentiation of human lung carcinoma cells by the interferon-alpha. *General Physiology and Biophysics*.

[11] McCormick C., Freshney R. I., Speirs V. (1995). Activity of interferon *α*, interleukin 6 and insulin in the regulation of differentiation in A549 alveolar carcinoma cells. *British Journal of Cancer*.

[12] Lepire M. L., Ziomek C. A. (1989). Preimplantation mouse embryos express a heat-stable alkaline phosphatase. *Biology of Reproduction*.

[13] Ginsburg M., Snow M. H. L., McLaren A. (1990). Primordial germ cells in the mouse embryo during gastrulation. *Development*.

[14] Pinkerton J. H., McKay D. G., Adams E. C., Hertig A. T. (1961). Development of the human ovary—a study using histochemical technics. *Obstetrics and Gynecology*.

[15] Stoop H., Honecker F., Cools M., de Krijger R., Bokemeyer C., Looijenga L. H. J. (2005). Differentiation and development of human female germ cells during prenatal gonadogenesis: an immunohistochemical study. *Human Reproduction*.

[16] Franke F. E., Pauls K., Rey R., Marks A., Bergmann M., Steger K. (2004). Differentiation markers of Sertoli cells and germ cells in fetal and early postnatal human testis. *Anatomy and Embryology*.

[17] Hustin J., Gillerot Y., Franchimont P., Collette J. (1990). Placental alkaline phosphatase in developing normal and abnormal gonads and in germ-cell tumours. *Virchows Archiv A*.

[18] Hustin J., Collette J., Franchimont P. (1987). Immunohistochemical demonstration of placental alkaline phosphatase in various states of testicular development and in germ cell tumours. *International Journal of Andrology*.

[19] MacGregor G. R., Zambrowicz B. P., Soriano P. (1995). Tissue non-specific alkaline phosphatase is expressed in both embryonic and extraembryonic lineages during mouse embryogenesis but is not required for migration of primordial germ cells. *Development*.

[20] Langer D., Ikehara Y., Takebayashi H., Hawkes R., Zimmermann H. (2007). The ectonucleotidases alkaline phosphatase and nucleoside triphosphate diphosphohydrolase 2 are associated with subsets of progenitor cell populations in the mouse embryonic, postnatal and adult neurogenic zones. *Neuroscience*.

[21] Kermer V., Ritter M., Albuquerque B., Leib C., Stanke M., Zimmermann H. (2010). Knockdown of tissue nonspecific alkaline phosphatase impairs neural stem cell proliferation and differentiation. *Neuroscience Letters*.

[22] Bossi M., Hoylaerts M. F., Millán J. L. (1993). Modifications in a flexible surface loop modulate the isozyme-specific properties of mammalian alkaline phosphatases. *Journal of Biological Chemistry*.

[23] Narisawa S., Hasegawa H., Watanabe K., Millan J. L. (1994). Stage-specific expression of alkaline phosphatase during neural development in the mouse. *Developmental Dynamics*.

[24] Hýžd'alová M., Hofmanová J., Pacherník J., Vaculová A., Kozubík A. (2008). The interaction of butyrate with TNF-alpha during differentiation and apoptosis of colon epithelial cells: Role of NF-kappaB activation. *Cytokine*.

[25] Barnard J. A., Warwick G. (1993). Butyrate rapidly induces growth inhibition and differentiation in HT-29 cells. *Cell Growth & Differentiation*.

[26] Hodin R. A., Meng S., Archer S., Tang R. (1996). Cellular growth state differentially regulates enterocyte gene expression in butyrate-treated HT-29 cells. *Cell Growth & Differentiation*.

[27] Hull W. E., Halford S. E., Gutfreund H., Sykes B. D. (1976). 31P nuclear magnetic resonance study of alkaline phosphatase: the role of inorganic phosphate in limiting the enzyme turnover rate at alkaline pH. *Biochemistry*.

[28] Zhang L., Balcerzak M., Radisson J. (2005). Phosphodiesterase activity of alkaline phosphatase in ATP-initiated Ca^+2^ and phosphate deposition in isolated chicken matrix vesicles. *The Journal of Biological Chemistry*.

[29] Cox R. P., Gilbert P., Griffin M. J. (1967). Alkaline inorganic pyrophosphatase activity of mammalian-cell alkaline phosphatase. *Biochemical Journal*.

[30] Georgatsos J. G. (1967). Specificity and phosphotransferase activity of purified placental alkaline phosphatase. *Archives of Biochemistry and Biophysics*.

[31] Stinson R. A., McPhee J. L., Collier H. B. (1987). Phosphotransferase activity of human alkaline phosphatases and the role of enzyme Zn^2+^. *Biochimica et Biophysica Acta—Protein Structure and Molecular*.

[32] Hofmann M. C., Millan J. L. (1993). Developmental expression of alkaline phosphatase genes; reexpression in germ cell tumours and in vitro immortalized germ cells. *European Urology*.

[33] Ulbright T. M. (2005). Germ cell tumors of the gonads: a selective review emphasizing problems in differential diagnosis, newly appreciated, and controversial issues. *Modern Pathology*.

[34] Fishman W. H., Inglis N. R., Green S. (1968). Immunology and biochemistry of Regan isoenzyme of alkaline phosphatase in human cancer. *Nature*.

[35] Li M., Gao J., Feng R. (2013). Generation of monoclonal antibody MS17-57 targeting secreted alkaline phosphatase ectopically expressed on the surface of gastrointestinal cancer cells. *PLoS ONE*.

[36] Goldsmith J. D., Pawel B., Goldblum J. R. (2002). Detection and diagnostic utilization of placental alkaline phosphatase in muscular tissue and tumors with myogenic differentiation. *The American Journal of Surgical Pathology*.

[37] Alpers D. H., Mahmood A., Engle M., Yamagishi F., DeSchryver-Kecskemeti K. (1994). The secretion of intestinal alkaline phosphatase (IAP) from the enterocyte. *Journal of Gastroenterology*.

[38] Mahmood A., Yamagishi F., Eliakim R., DeSchryver-Kecskemeti K., Gramlich T. L., Alpers D. H. (1994). A possible role for rat intestinal surfactant-like particles in transepithelial triacylglycerol transport. *The Journal of Clinical Investigation*.

[39] Shao J.-S., Engle M., Xie Q. (2000). Effect of tissue non-specific alkaline phosphatase in maintenance of structure of murine colon and stomach. *Microscopy Research and Technique*.

[40] Narisawa S., Huang L., Iwasaki A., Hasegawa H., Alpers D. H., Millán J. L. (2003). Accelerated fat absorption in intestinal alkaline phosphatase knockout mice. *Molecular and Cellular Biology*.

[41] Mizumori M., Ham M., Guth P. H., Engel E., Kaunitz J. D., Akiba Y. (2009). Intestinal alkaline phosphatase regulates protective surface microclimate pH in rat duodenum. *The Journal of Physiology*.

[42] Bates J. M., Akerlund J., Mittge E., Guillemin K. (2007). Intestinal alkaline phosphatase detoxifies lipopolysaccharide and prevents inflammation in zebrafish in response to the gut microbiota. *Cell Host and Microbe*.

[43] Goldberg R. F., Austen W. G., Zhang X. (2008). Intestinal alkaline phosphatase is a gut mucosal defense factor maintained by enteral nutrition. *Proceedings of the National Academy of Sciences of the United States of America*.

[44] Suzuki A., Guicheux J., Palmer G. (2002). Evidence for a role of p38 MAP kinase in expression of alkaline phosphatase during osteoblastic cell differentiation. *Bone*.

[45] Suzuki A., Palmer G., Bonjour J.-P., Caverzasio J. (1999). Regulation of alkaline phosphatase activity by p38 MAP kinase in response to activation of Gi protein-coupled receptors by epinephrine in osteoblast-like cells. *Endocrinology*.

[46] Rey A., Manen D., Rizzoli R., Ferrari S. L., Caverzasio J. (2007). Evidences for a role of p38 MAP kinase in the stimulation of alkaline phosphatase and matrix mineralization induced by parathyroid hormone in osteoblastic cells. *Bone*.

[47] Caverzasio J., Manen D. (2007). Essential role of Wnt3a-mediated activation of mitogen-activated protein kinase p38 for the stimulation of alkaline phosphatase activity and matrix mineralization in C3H10T1/2 mesenchymal cells. *Endocrinology*.

[48] Allen M., Svensson L., Roach M., Hambor J., McNeish J., Gabel C. A. (2000). Deficiency of the stress kinase p38*α* results in embryonic lethality: characterization of the kinase dependence of stress responses of enzyme-deficient embryonic stem cells. *The Journal of Experimental Medicine*.

[49] Kim J. M., White J. M., Shaw A. S., Sleckman B. P. (2005). MAPK p38*α* is dispensable for lymphocyte development and proliferation. *Journal of Immunology*.

[50] Tesar P. J., Chenoweth J. G., Brook F. A. (2007). New cell lines from mouse epiblast share defining features with human embryonic stem cells. *Nature*.

[51] Brons I. G. M., Smithers L. E., Trotter M. W. B. (2007). Derivation of pluripotent epiblast stem cells from mammalian embryos. *Nature*.

[52] Narisawa S., Fröhlander N., Millán J. L. (1997). Inactivation of two mouse alkaline phosphatase genes and establishment of a model of infantile hypophosphatasia. *Developmental Dynamics*.

[53] Buehr M. (1997). The primordial germ cells of mammals: some current perspectives. *Experimental Cell Research*.

[54] Shimada N., Yamada K., Tanaka T. (2001). Alterations of gene expression in endoderm differentiation of F9 teratocarcinoma cells. *Molecular Reproduction and Development*.

[55] Zwaka T. P., Thomson J. A. (2005). A germ cell origin of embryonic stem cell?. *Development*.

[56] Mulivor R. A., Plotkin L. I., Harris H. (1978). Differential inhibition of the products of the human alkaline phosphatase loci. *Annals of Human Genetics*.

[57] van Belle H. (1976). Alkaline phosphatase. I. Kinetics and inhibition by levamisole of purified isoenzymes from humans. *Clinical Chemistry*.

[58] Komoda T., Hokari S., Sonoda M., Sakagishi Y., Tamura T. (1982). L-phenylalanine inhibition of human alkaline phosphatases with p-nitrophenyl phosphate as substrate. *Clinical Chemistry*.

[59] Lin C. W., Sie H. G., Fishman W. H. (1971). L-tryptophan. A non-allosteric organ-specific uncompetitive inhibitor of human placental alkaline phosphatase. *Biochemical Journal*.

[60] Doellgast G. J., Fishman W. H. (1976). L leucine a specific inhibitor of a rare human placental alkaline phosphatase phenotype. *Nature*.

[61] Fishman W. H., Sie H.-G. (1970). L-Homoarginine; an inhibitor of serum “bone and liver” alkaline phosphatase. *Clinica Chimica Acta*.

[62] Brunel C., Cathala G. (1972). Imidazole: aan inhibitor of l-phenylalanine-insensitive alkaline phosphatases of tissues other than intestine and placenta. *Biochimica et Biophysica Acta—Enzymology*.

[63] Nouwen E. J., Hendrix P. G., Dauwe S., Eerdekens M. W., de Broe M. E. (1987). Tumor markers in the human ovary and its neoplasms. A comparative immunohistochemical study. *The American Journal of Pathology*.

[64] O'Connor M. D., Kardel M. D., Iosfina I. (2008). Alkaline phosphatase-positive colony formation is a sensitive, specific, and quantitative indicator of undifferentiated human embryonic stem cells. *Stem Cells*.

[65] Pera M. F., Reubinoff B., Trounson A. (2000). Human embryonic stem cells. *Journal of Cell Science*.

[66] Ginis I., Luo Y., Miura T. (2004). Differences between human and mouse embryonic stem cells. *Developmental Biology*.

[67] Andäng M., Hjerling-Leffler J., Moliner A. (2008). Histone H2AX-dependent GABA(A) receptor regulation of stem cell proliferation. *Nature*.

[68] Yamanaka S., Takahashi K. (2006). Induction of pluripotent stem cells from mouse fibroblast cultures. *Tanpakushitsu Kakusan Koso: Protein, Nucleic Acid, Enzyme*.

[69] Loh Y.-H., Wu Q., Chew J.-L. (2006). The Oct4 and Nanog transcription network regulates pluripotency in mouse embryonic stem cells. *Nature Genetics*.

[70] González F., Boué S., Belmonte J. C. I. (2011). Methods for making induced pluripotent stem cells: reprogramming à la carte. *Nature Reviews Genetics*.

[71] Williams R. L., Hilton D. J., Pease S. (1988). Myeloid leukaemia inhibitory factor maintains the developmental potential of embryonic stem cells. *Nature*.

[72] Rohwedel J., Guan K., Wobus A. M. (1999). Induction of cellular differentiation by retinoic acid in vitro. *Cells Tissues Organs*.

[73] Mark M., Ghyselinck N. B., Chambon P. (2009). Function of retinoic acid receptors during embryonic development. *Nuclear Receptor Signaling*.

[74] Mummery C. L., Feyen A., Freund E., Shen S. (1990). Characteristics of embryonic stem cell differentiation: a comparison with two embryonal carcinoma cell lines. *Cell Differentiation and Development*.

[75] Pacherník J., Ešner M., Bryja V., Dvořák P., Hampl A. (2002). Neural differentiation of mouse embryonic stem cells grown in monolayer. *Reproduction Nutrition Development*.

[76] Pacherník J., Bryja V., Ešner M., Kubala L., Dvořák P., Hampl A. (2005). Neural differentiation of pluripotent mouse embryonal carcinoma cells by retinoic acid: inhibitory effect of serum. *Physiological Research*.

[77] Kotasová H., Veselá I., Kučera J. (2012). Phosphoinositide 3-kinase inhibition enables retinoic acid-induced neurogenesis in monolayer culture of embryonic stem cells. *Journal of Cellular Biochemistry*.

[78] Gianni' M., Studer M., Carpani G., Terao M., Garattini E. (1991). Retinoic acid induces liver/bone/kidney-type alkaline phosphatase gene expression in F9 teratocarcinoma cells. *The Biochemical Journal*.

[79] Scheibe R. J., Moeller-Runge I., Mueller W. H. (1991). Retinoic acid induces the expression of alkaline phosphatase in P19 teratocarcinoma cells. *The Journal of Biological Chemistry*.

[80] Preclíková H., Bryja V., Pacherník J., Krejčí P., Dvořák P., Hampl A. (2002). Early cycling-independent changes to p27, cyclin D2, and cyclin D3 in differentiating mouse embryonal carcinoma cells. *Cell Growth and Differentiation*.

[81] Bryja V., Pacherník J., Vondráček J. (2008). Lineage specific composition of cyclin D-CDK4/CDK6-p27 complexes reveals distinct functions of CDK4, CDK6 and individual D-type cyclins in differentiating cells of embryonic origin. *Cell Proliferation*.

[82] Bryja V., Pacherník J., Souček K., Horvath V., Dvořák P., Hampl A. (2004). Increased apoptosis in differentiating p27-deficient mouse embryonic stem cells. *Cellular and Molecular Life Sciences*.

[83] Nayernia K., Nolte J., Michelmann H. W. (2006). In vitro-differentiated embryonic stem cells give rise to male gametes that can generate offspring mice. *Developmental Cell*.

[84] Elliott A. M., de Miguel M. P., Rebel V. I., Donovan P. J. (2007). Identifying genes differentially expressed between PGCs and ES cells reveals a role for CREB-binding protein in germ cell survival. *Developmental Biology*.

[85] Pikarsky E., Sharir H., Ben-Shushan E., Bergman Y. (1994). Retinoic acid represses Oct-3/4 gene expression through several retinoic acid-responsive elements located in the promoter-enhancer region. *Molecular and Cellular Biology*.

[86] Kim J. S., Kim B. S., Kim J., Park C.-S., Chung I. Y. (2010). The phosphoinositide-3-kinase/Akt pathway mediates the transient increase in Nanog expression during differentiation of F9 cells. *Archives of Pharmacal Research*.

[87] Hallmann D., Trümper K., Trusheim H. (2003). Altered signaling and cell cycle regulation in embryonal stem cells with a disruption of the gene for phosphoinositide 3-kinase regulatory subunit p85*α*. *The Journal of Biological Chemistry*.

[88] Paling N. R. D., Wheadon H., Bone H. K., Welham M. J. (2004). Regulation of embryonic stem cell self-renewal by phosphoinositide 3-kinase-dependent signaling. *The Journal of Biological Chemistry*.

[89] Rodda D. J., Chew J.-L., Lim L.-H. (2005). Transcriptional regulation of Nanog by OCT4 and SOX2. *Journal of Biological Chemistry*.

[90] Pan G., Li J., Zhou Y., Zheng H., Pei D. (2006). A negative feedback loop of transcription factors that controls stem cell pluripotency and self-renewal. *The FASEB Journal*.

[91] Yamaguchi S., Kurimoto K., Yabuta Y. (2009). Conditional knockdown of Nanog induces apoptotic cell death in mouse migrating primordial germ cells. *Development*.

[92] Heine P., Braun N., Heilbronn A., Zimmermann H. (1999). Functional characterization of rat ecto-ATPase and ecto-ATP diphosphohydrolase after heterologous expression in CHO cells. *European Journal of Biochemistry*.

[93] Jaiswal R. K., Jaiswal N., Bruder S. P., Mbalaviele G., Marshak D. R., Pittenger M. F. (2000). Adult human mesenchymal stem cell differentiation to the osteogenic or adipogenic lineage is regulated by mitogen-activated protein kinase. *The Journal of Biological Chemistry*.

[94] Bühring H.-J., Treml S., Cerabona F., De Zwart P., Kanz L., Sobiesiak M. (2009). Phenotypic characterization of distinct human bone marrow-derived MSC subsets. *Annals of the New York Academy of Sciences*.

[95] Gargett C. E., Masuda H. (2010). Adult stem cells in the endometrium. *Molecular Human Reproduction*.

[96] Sobiesiak M., Sivasubramaniyan K., Hermann C. (2010). The mesenchymal stem cell antigen MSCA-1 is identical to tissue non-specific alkaline phosphatase. *Stem Cells and Development*.

[97] Kim Y. H., Yoon D. S., Kim H. O., Lee J. W. (2012). Characterization of different subpopulations from bone marrow-derived mesenchymal stromal cells by alkaline phosphatase expression. *Stem Cells and Development*.

[98] Kenmotsu M., Matsuzaka K., Kokubu E., Azuma T., Inoue T. (2010). Analysis of side population cells derived from dental pulp tissue. *International Endodontic Journal*.

